# Adaptive Ship Detection for Single-Look Complex SAR Images Based on SVWIE-Noncircularity Decomposition

**DOI:** 10.3390/s18103293

**Published:** 2018-09-30

**Authors:** Yu-Huan Zhao, Peng Liu

**Affiliations:** Key Laboratory for Information Science of Electromagnetic Waves, Ministry of Education, Fudan University, Shanghai 200433, China; 16210720110@fudan.edu.cn

**Keywords:** ship detection, synthetic aperture radar, SVWIE-noncircularity decomposition

## Abstract

In this paper, we present an adaptive ship detection method for single-look complex synthetic aperture radar (SAR) images. First, noncircularity is analyzed and adopted in ship detection task; besides, similarity variance weighted information entropy (SVWIE) is proposed for clutter reduction and target enhancement. According to the analysis of scattering of SVWIE and noncircularity, SVWIE-noncircularity (SN) decomposition is developed. Based on the decomposition, two components, the high-noncircularity SVWIE amplitude (*h*) and the low-noncircularity SVWIE amplitude (*l*), are obtained. We demonstrate that ships and clutter in SAR images are different for *h* detector and *h* detector can be effectively used for ship detection. Finally, to extract ships from the background, the generalized Gamma distribution (GΓD) is used to fit *h* statistics of clutter and the constant false alarm rate (CFAR) is utilized to choose an adaptive threshold. The performance of the proposed method is demonstrated on HH polarization of Alos-2 images. Experimental results show that the proposed method can accurately detect ships in complex background, i.e., ships are close to small islands or with strong noise.

## 1. Introduction

Synthetic aperture radar (SAR) is a powerful remote sensing technology, providing valuable information of the Earth surface with 24-hour all-weather sensing capability [1,2,3]. As one of the most important tasks in maritime surveillance, ship detection in SAR imagery has received much attention [4,5]. Since decades of research for ship detection, scads of algorithms have been studied with rather good results. The most frequently used ship detection algorithm is the intensity-based constant false alarm rate (CFAR) method with a suitable probability density function (PDF) for the background clutter model [6,7,8]. Other methods are based on the polarimetric analysis [9,10,11] and multi-look cross-correlation [12,13], etc. However, when ships are in heavy background noise, the task becomes challenging.

In recent studies, various studies have been conducted to enhance ships and inhibit noise in order to improve detectability of ships in complex situations. A method based on the variance weighted information entropy (VWIE) has been proposed [14], which could suppress the background noise and enhance regions of ship from various circumstances without prior knowledge. Wang tacked on multi-scale information into VWIE to deal with the problem of different size of ships [15]. A combination of VWIE and local contrast information has been proposed to detect ships from complex background [16]. However, these methods only focus on utilizing intensity of SAR data and discarding the imaginary part. Therefore, when the backscattering from sea clutter or islands are close to those of ships, the detection ability will be weakened.

With increasing SAR resolution in recent years, there is a growing interest in the information kept in the complex-valued SAR data rather than ignoring the imaginary part. Phase information is a significant element in many SAR applications, such as SAR interferometry [17]. Noncircularity is one of the statistical characteristics that contains the phase information of complex data, which describes the distribution difference between the real and imaginary parts [18,19,20]. Noncircularity is widely employed in many areas, such as smart grid and magnetic resonance imaging [21,22]. Wu employed this parameter to distinguish man-made targets from natural background in SAR images [23,24]. Leng found that ships have higher noncircularity than the ambient ocean, which shows great potential in ship detection task in complex background [25]. For high resolution SAR imagery, noncircularity would perform better because fewer scatterers are contained in a single pixel [26].

Inspired by the advantages of VWIE and noncircularity, we propose a novel method aiming to detect ships in complex background. The remainder of this paper is organized as follows. Section 2 introduces the theories of noncircularity and the proposed similarity variance weighted information entropy (SVWIE). Accordingly, the derivation of the SVWIE-Noncircularity (SN) decomposition, development of *h* detector and corresponding CFAR method are presented in details. Experiments and discussions are described in Section 3. Section 4 presents the conclusions of this paper.

## 2. The Proposed Method

### 2.1. Algorithm Overview

The performance of ship detection algorithms in SAR images largely depends on weather and sea surface conditions [4]. With well-developed high waves, high velocity of near-surface wind, and convective structures, ship detection becomes rather difficult due to the raised backscattering from the surroundings. Besides, it is a challenging task to detect ships when they are close to islands. It is necessary to reduce the influence of the obstruction. The proposed algorithm is aimed at detecting ships in the complex situations which cannot be easily done only by the utilization of intensity.

In this paper, noncircularity level, describing the differences between the real and imaginary parts of a SAR image [24], is adopted for the ship detection. Compared with classic ship detection algorithms which are basically based on the intensity, the new method takes advantage of both the intensity and phase information.

**Figure 1:**
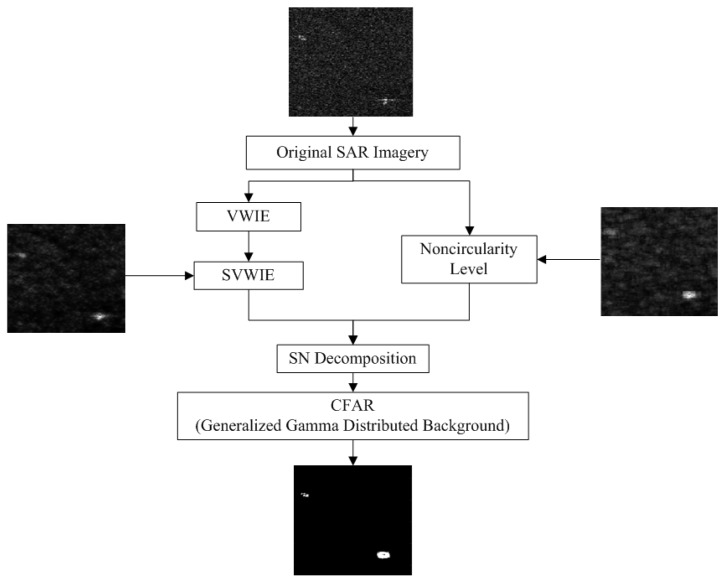
Flowchart of the proposed method.

The flowchart of proposed algorithm is shown in Figure 1. First, with similarity measure added, SVWIE is presented. Relied on SVWIE and noncircularity level, SN decomposition is developed. SN decomposition based *h* detector is used to extract ships from complex background adaptively using CFAR; besides, the generalized Gamma distribution (GΓD) is found suitable for characterize the statistics of *h* detector.

The originalities of the proposed method are:

- For the first time, noncircularity level is adopted in ship detection, which makes use of phase information and is effective for reducing the interference of strong backscattering;
- The SVWIE is proposed, which can further strengthen ships and suppress clutter;
- The *h* detector is developed based on a novel SN decomposition to extract ships from complex background;
- Lastly, the comprehensive ship detection framework for complex background has been designed.

### 2.2. Why Should We Take Noncircularity into Account for Ship Detection?

In this subsection, the concept of noncircularity is introduced. Besides, an example is conducted to show potential advantages of noncircularity to detect ships in the complex background. Further consideration for noncircularity is presented.

#### 2.2.1. Noncircularity

For complex-valued signals, noncircularity is a property of PDF, indicating the distribution consistency between the real and imaginary parts. For a complex random variable $Z=Z_{R}+jZ_{I}$, where $Z_{R}$ indicates the real part and $Z_{I}$ denotes the imaginary part, if *Z* and $Z\cdot e^{j\theta }$ (θ represents a phase angle) have the same PDF, the PDF is a function of the magnitude ${\left|Z\right|}^{2}=Z\cdot Z^{*}=Z_{R}^{2}+Z_{I}^{2}$, indicating that the distribution of the complex random is rotationally invariant [18]. Therefore, for circularity plots in the complex plane in the case, the constant contours are circles.

To measure the noncircularity quantitatively, the noncircularity level is given as [27]

(1) $$T_{non-cir}=\left|\frac{\sum_{i=1}^{N}Z_{i}^{2}}{\sum_{i=1}^{N}{\left|Z_{i}\right|}^{2}}\right|,$$

where $Z_{i}$ is a complex number from *N* samples within a window. Window size is an important parameter, since $T_{non-cir}$ is statistical information. Note that the larger window size is used, the more samples are included, the more accurate the statistical distribution is estimated, and the more robust $T_{non-cir}$ is [24]. The denominator in Equation (1) is $\sum_{i=1}^{N}{\left|Z_{i}\right|}^{2}$, denoting the sum of pixels’ intensity within a window. This is means that $T_{non-cir}$ complements intensity and can perform different characteristics from intensity. $T_{non-cir}$ is limited to the interval [0, 1]. Within the limit, higher $T_{non-cir}$ means more determinate scatterer in SAR images [23]. When scattering is random, the real and imaginary parts obey the same zero-mean Gaussian distribution, indicating circular. However, for determinate scattering, the corresponding distributions of the real and imaginary parts are different, which means that the complex data are noncircular. Therefore, $T_{non-cir}$ is a favorable parameter for ship detection.

#### 2.2.2. The Effectiveness of Noncircularity

**Figure 2:**
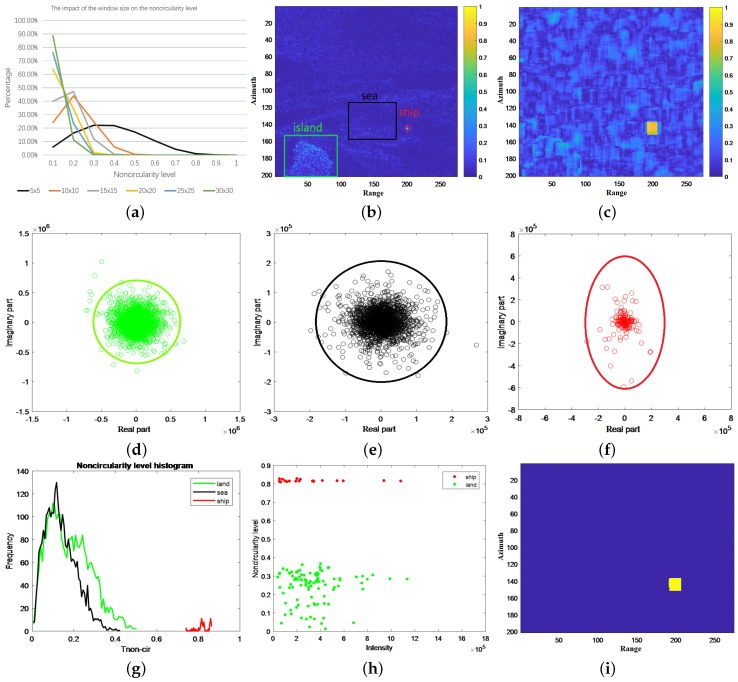
Noncircularity level: (**a**) Impact of the window size on $T_{non-cir}$; (**b**) Original SAR image chip; (**c**) $T_{non-cir}$ image; (**d**) Circularity plot of the island; (**e**) Circularity plot of the ambient sea; (**f**) Circularity plot of the ship; (**g**) Histogram of $T_{non-cir}$ of the land, sea and ship regions; (**h**) Intensity-$T_{non-cir}$ plane; (**i**) Thresholding result.

The effectiveness of $T_{non-cir}$ is illustrated in the following example. The window size is fixed to 15 × 15 (We compute corresponding $T_{non-cir}$ under the circular hypothesis with different window sizes, shown in Figure 2a. With the increase of the window size, $T_{non-cir}$ drops rapidly. However, there is a trade-off between $T_{non-cir}$ and size of detection results, both related to the window size. Therefore, we choose a window with a size of 15 × 15.). An ALOS-2 HH polarized SAR image chip (200 × 275 pixels, resolution: 4.642 m × 4.29 m (range × azimuth)) where there is a ship near an island is shown in Figure 2b. The ship, ambient sea, island regions are marked by red, black and green rectangles, respectively. By using Equation (1), $T_{non-cir}$ image, indicating noncircularity level of the original SAR image, is presented in Figure 2c, where the ship region is greatly enhanced and the island region is weakened. The circularity plots in complex plane for the island, ambient sea and ship regions are shown in Figure 2d–f, respectively. The contours of circularity plots of the island and sea are close to circles with approximately 95% of the points lying inside or on the circles, indicating low $T_{non-cir}$. However, the contour of circularity plot of the ship is almost an ellipse containing all the points, which means high $T_{non-cir}$. The histogram of $T_{non-cir}$ of the island, sea and ship regions is shown in Figure 2g. It is clear that there is a notch between the $T_{non-cir}$ of the island/sea and ship regions. Figure 2h is the intensity-$T_{non-cir}$ plane, from which we can see that although the ship cannot be distinguished from the island only using intensity of SAR data, the ship and island regions can be separated from $T_{non-cir}$ perspective. If we set a threshold of $T_{non-cir}$ at 0.6, the island can be erased and the region of ship is located, as shown in Figure 2i.

Based on Figure 2, several observations can be made:

- Although the island and sea regions have different backscattering, $T_{non-cir}$ of them is almost the same. Besides, although the ship and island regions have almost the same backscattering, $T_{non-cir}$ of them has great difference. Thus, $T_{non-cir}$ can help to distinguish ships from complex background when the background has almost the same backscattering as ships, which is difficult to be done only by the image intensity.
- $T_{non-cir}$ of ship region does not definitely equal 1 and $T_{non-cir}$ of island/sea regions does not absolutely equal 0. Therefore, $T_{non-cir}$ is not always a perfect parameter to extract ship regions and needs modification.
- Since the calculation of $T_{non-cir}$ needs window sliding, $T_{non-cir}$ image turns out to be rectangular and the ship region would be enlarged a bit depending on the window size.

#### 2.2.3. Further Consideration of Noncircularity

**Figure 3:**
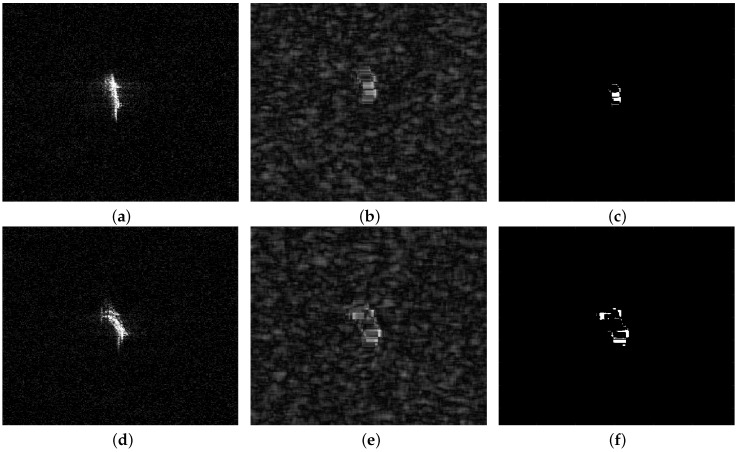
Detection results by noncircularity level alone: (**a**) Original SAR image; (**b**) $T_{non-cir}$ image of Figure 3a; (**c**) Detection result by $T_{non-cir}$ alone; (**d**) Original SAR image; (**e**) $T_{non-cir}$ image of Figure 3d; (**f**) Detection result by $T_{non-cir}$ alone.

Despite aforementioned advantages of noncircularity, there remain weak points if only employing noncircularity to detect ships. Noncircularity alone is insufficient for ship detection, because noncircularity level of large ships shows inconsistency and the detection results will become intermittent, which is a disadvantage. Figure 3 shows detection results by noncircularity alone, from which we can see that although ship areas can be also detected, the detection results are not good, especially for the aspect of the shape of ships.

Moreover, noncircularity disregards other factors which are crucial to detect ships, such as gray information, etc. Therefore, rather than use noncircularity level alone, it is necessary to combine noncircularity with other features, i.e., SVWIE, to improve ship detection performance.

### 2.3. Similarity Variance Weighted Information Entropy (SVWIE)

Information entropy reflects the complex degree of the gray values of an image [28]. For a SAR image patch with 256 gray levels, the information entropy is defined as [29]

(2) $$E=-\sum_{i=0}^{255}p_{i}\log\left(p_{i}\right),$$

where $p_{i}$ is the probability of the *i*th gray level. Note that let $p_{i}\log\left(p_{i}\right)=0$ when $p_{i}=0$. Although the information entropy indicates how much information contains in an image, it cannot reflect the contribution of high gray value that is also of great significance as ships always have high gray values. Therefore, the gray value, which is derived from intensity of SAR data, is added to Equation (2) as [15]

(3) $$H\left(m,n\right)=-\sum_{i=0}^{255}\left|I_{i}-\bar{I}\right|p_{i}\log\left(p_{i}\right),$$

where $H\left(m,n\right)$ is the VWIE at pixel (m,n), $I_{i}$ is the gray value of pixels in the local window of the pixel (m,n) and $\bar{I}$ is the mean gray value in the local window. *H* contains information of complexity of an image and gray information against surroundings. However, it fails to take information of similarity among pixels into account. Similarity measure is crucial for detection, since neighbor pixels with similar intensity are more likely to belong to the same category.

There are two main factors influencing similarity measure, intensity value and distance. To simplify the difference of intensity value, SAR imagery is quantified into 16 gray levels to compute intensity similarity. The intensity similarity of two pixels (m,n) and (p,q) is defined as follows [30]:

(4) $$D=\left|A-B\right|,$$

where A and B represent the corresponding levels of pixel (m,n) and pixel (p,q). Besides, the distance similarity of two pixels (m,n) and (p,q) is given by Euclidean distance:

(5) $$R=\sqrt{{\left(m-p\right)}^{2}+{\left(n-q\right)}^{2}}.$$

The similarity measure of pixel (m,n) to its surrounding pixels in a window of size *T* is

(6) $$S\left(m,n\right)=\sum_{\begin{array}{c} \left|m-p\right|\leq \frac{T}{2}\\ \left|n-q\right|\leq \frac{T}{2} \end{array}}\frac{I\left(p,q\right)\times \alpha^{-D}}{k\times R^{2}},$$

where α is a constant that controls attenuation speed of intensity similarity, and *k* is a constant that balances the weight between intensity and distance similarity. We choose α=1.2,k=1 for ALOS-2 SAR imagery in our case.

VWIE can be modified by similarity measure and written as follows:

(7) $$SH\left(m,n\right)=S\left(m,n\right)\times H\left(m,n\right),$$

where $SH\left(m,n\right)$ is the SVWIE at pixel (m,n). From Equation (7), SVWIE can be interpreted as following two ways. On one hand, VWIE is weighted by similarity measure value. Therefore, VWIE with low similarity measure which means high possibility of clutter, can be weakened, because abnormal large intensity value has poor similarity measure. Meanwhile, VWIE with high similarity measure which is usually ship targets, will be enhanced. On the other hand, SVWIE can be seen as a multiple combination of features which are gray information, similarity measure and complexity information. Each of the features strengthens ships and suppresses clutter.

### 2.4. SVWIE-Noncircularity Decomposition

Target decomposition is a well-established method to extract the scattering difference among different types of objects [9]. In order to detect ships with few false alarms, $T_{non-cir}$ should be considered as it is an effective parameter to reduce the interference of islands and strong sea clutter. According to the contribution of $T_{non-cir}$ in response, based on Equations (1) and (7), SVWIE can be decomposed into the following two components:

(8) $$SH^{2}=h^{2}+l^{2},$$

with

(9) $$\begin{array}{c} h=\sqrt{SH\times T_{non-cir}},\;l=\sqrt{SH\times \left(1-T_{non-cir}\right)}, \end{array}$$

where *h* and *l* represent the high-noncircularity SVWIE amplitude and the low-noncircularity SVWIE amplitude, respectively. The SN decomposition is effective in ship detection for the following two reasons. From the scattering perspective, ships exhibit a larger coherent scattering. Ships have complicated superstructure and metallic material, which results in a much stronger backscattering power. The pixels representing ships in SAR images, therefore, have a higher SVWIE value than clutter [14,30]. Besides, because of the structure of ships, e.g., deck, mast and containers, the complex vectors from ships received by the SAR sensor are generally more ordered, i.e., noncircular, but the complex vectors from the ocean/islands without man-made targets are generally random, i.e., circular [31]. Thus, ships have higher $T_{non-cir}$ [23,25]. As *h* detector is the product of SVWIE and $T_{non-cir}$, ships will have larger *h* values than clutter. From feature utilization prospective, *h* detector can be regarded as a mutual reinforcement of SVWIE and $T_{non-cir}$, with the former relevant to backscattering power and the latter related to noncircularity. As we mentioned in Section 2.2.3, detection results only by noncircularity alone may become intermittent and incomplete, it is important to make up for this shortcoming. SVWIE is an excellent choice, because it can enhance the whole ship regions. Moreover, noncircularity presents different characteristics from intensity. SVWIE is a combination parameter of intensity information with similarity measure and complexity information. Therefore, Noncircularity and SVWIE complement each other well.

### 2.5. Adaptive Thresholding

In order to obtain an adaptive threshold for ship detection, a proper statistical model of clutter must be adopted. Here the GΓD is chosen because it is a versatile model for describing the statistical behavior. The PDF can be expressed as [32]

(10) $$p\left(x\right)=\frac{\left|\upsilon \right|\kappa^{\kappa }}{\sigma \Gamma \left(\kappa \right)}{\left(\frac{x}{\sigma }\right)}^{\kappa v-1}\times \exp\left\{-\kappa {\left(\frac{x}{\sigma }\right)}^{\upsilon }\right\},\sigma ,\left|\upsilon \right|,\kappa ,x>0,$$

where $\Gamma \left(x\right)$ is the Gamma function, σ, κ and υ refer to the scale, power, and shape parameters. The GΓD contains many famous laws used for modeling SAR images, such as Rayleigh (υ=2 and κ=1), gamma (υ=1), Weibull(κ=1) and exponential (υ=1 and κ=1) [9]. The CFAR threshold based on the GΓD can be deduced as

(11) $$T=\left\{\begin{array}{c} \sigma {\left(\frac{1}{\kappa }\Gamma^{-1}\left(1-p_{fa},\kappa \right)\right)}^{\frac{1}{\upsilon }},\upsilon >0\\ \sigma {\left(\frac{1}{\kappa }\Gamma^{-1}\left(p_{fa},\kappa \right)\right)}^{\frac{1}{\upsilon }},\upsilon <0 \end{array}\right.$$

where $p_{fa}$ is the value of false alarm probability and $\Gamma^{-1}$ is the inverse Gamma function.

## 3. Experiments and Results

To evaluate the performance of the theoretical analysis in Section 2, the proposed method is validated by real SAR images from ALOS-2 satellite. The polarization is HH mode. The resolution is 4.642 m × 4.29 m (range × azimuth). The fitting abilities of the GΓD for *h* statistics used for CFAR ship detection is evaluated. Besides, we assess the effectiveness of *h* detector compared with SVWIE and $T_{non-cir}$. Finally, the proposed method performances are compared with those of VWIE [14], SVWIE, GΓD-CFAR [33] and the state-of-the-art truncated satistics (TS)-CFAR [8].

### 3.1. The Effectiveness of GΓD for *h* Detector

**Figure 4:**
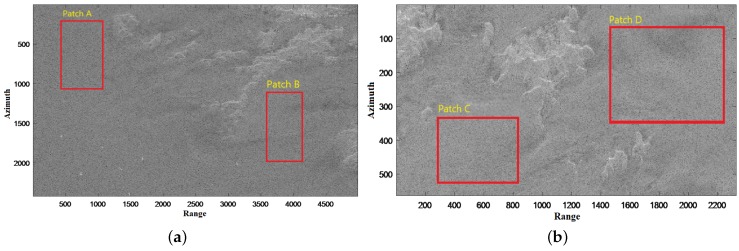
Test scenes for GΓD fitting sea clutter: (**a**) Scene 1. (**b**) Scene 2.

**Figure 5:**
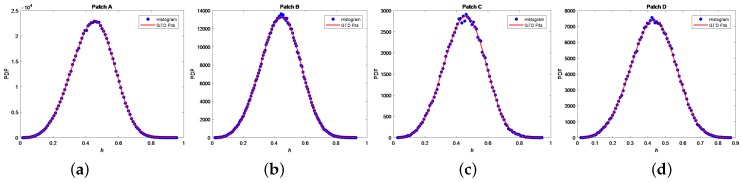
Fitting experiments using the GΓD in patches A, B, C and D: (**a**–**d**) fitting results of *h* statistics for Patches A, B, C and D, respectively.

We investigate appropriateness of the GΓD modeling for *h* statistics. Four sea clutter regions, namely patches A and B from Figure 4a, as well as patches C and D from Figure 4b, are extracted. The GΓD is utilized to fit histograms of the four patches of *h*. Figure 5 shows the results of the fitting experiments. To quantitatively assess the fitting results, the Kullback-Leibler (KL) distance [34] is employed, and the KL values are shown in Table 1. It is obvious that the GΓD fully agrees with *h* statistics because the order of magnitute is $10^{-3}$, which is sufficiently small.

### 3.2. The Effectiveness of *h* Detector

**Figure 6:**
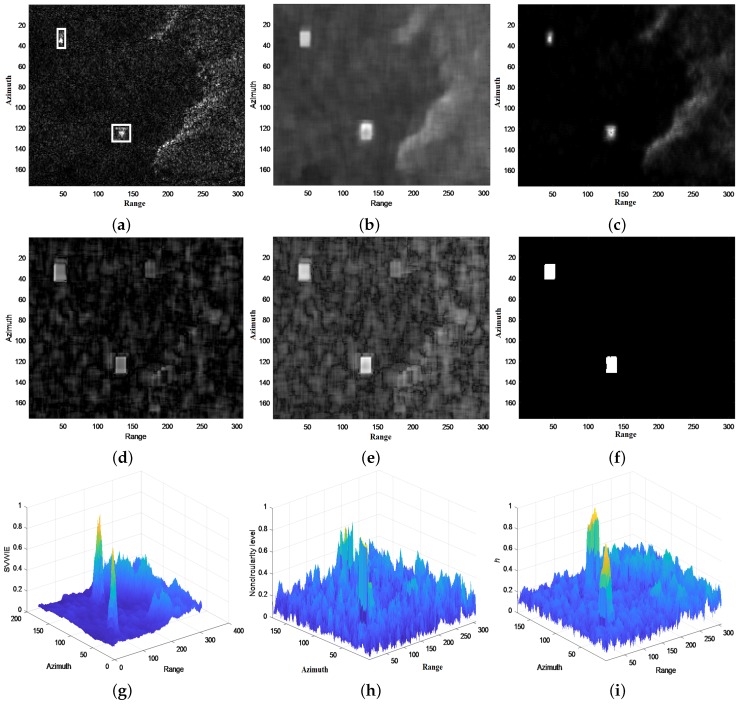
Effectiveness of *h* detector: (**a**) Original SAR image; (**b**) VWIE image; (**c**) SVWIE image; (**d**) $T_{non-cir}$ image; (**e**) *h* image; (**f**) Detection result; (**g**) 3-D display of the SVWIE image for the interval [0, 1]; (**h**) 3-D display of the $T_{non-cir}$ image for the interval [0, 1]; (**i**) 3-D display of the *h* image for the interval [0, 1].

To validate the effectiveness of *h* detector, which is based on SN decomposition, an example is employed in Figure 6. It can be seen from Figure 6a, there are two ships near the coast in the complex background. The VWIE image is shown in Figure 6b, with both ships and the coast region enhanced. Figure 6c is the SVWIE image, which suppresses the background clutter and coast region. However, the coast region is still disrupting for detection. The $T_{non-cir}$ image is shown in Figure 6d. $T_{non-cir}$ helps highlight the ship regions and decrease the effect the coast region, which contributes to prevent from detecting the coast as ships. Figure 6e is the *h* image. Compared to SVWIE image, *h* image enhances ships and largely reduces the influence of the coast region. Besides, compared to $T_{non-cir}$ image, *h* image further strengthens ships and suppresses background sea clutter. Detection result is shown in Figure 6f, which correctly extracts two regions of ship without any alarms. 3-D displays of the SVWIE, $T_{non-cir}$ and *h* images for the interval [0, 1] are provided in Figure 6g–i, respectively, from which we can see more clearly the enhancement of ships and suppression of clutter in the *h* image compared to the SVWIE and $T_{non-cir}$ images.

Based on Figure 6, several conclusions can be made:

- SVWIE works better at suppressing clutter than VWIE, as shown in Figure 6b,c.
- *h* detector is effective for reducing clutter and detecting ships, as shown Figure 6e,f, as it combines advantages of SVWIE and noncircularity.
- Compared to ships in Figure 6a, the ships in *h* image would be enlarged a bit because of window sliding. Solving this problem will be included in our future work.

### 3.3. Comparisons of Different Methods

To evaluate the proposed method, the detection results are compared with those obtained by VWIE, SVWIE, GΓD-CFAR and TS-CFAR. The true targets, confirmed by the AIS (Automatic Identification System) information, are marked by white rectangles. And the false targets are marked by red circles.

**Figure 7:**
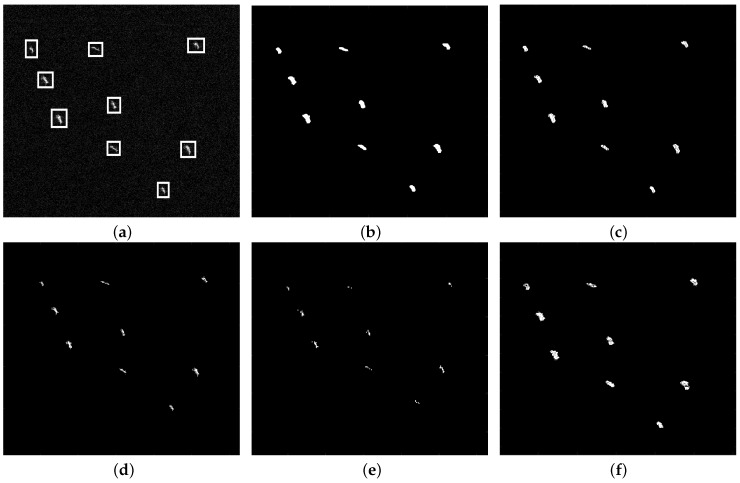
The homogenous scene: (**a**) Original SAR image; (**b**) Detection result of VWIE; (**c**) Detection result of SVWIE; (**d**) Detection result of GΓD-CFAR; (**e**) Detection result of TS-CFAR; (**f**) Detection result of the proposed method.

Figure 7a is a homogeneous background image containing nine ships (2100 × 1200 pixels). Figure 7b–f show detection results of VWIE, SVWIE, GΓD-CFAR, TS-CFAR and the proposed method, respectively. Because of the homogenous situation, i.e., high signal-to-clutter ratio (SCR), all methods can highlight nine ships correctly without false or missing alarms.

**Figure 8:**
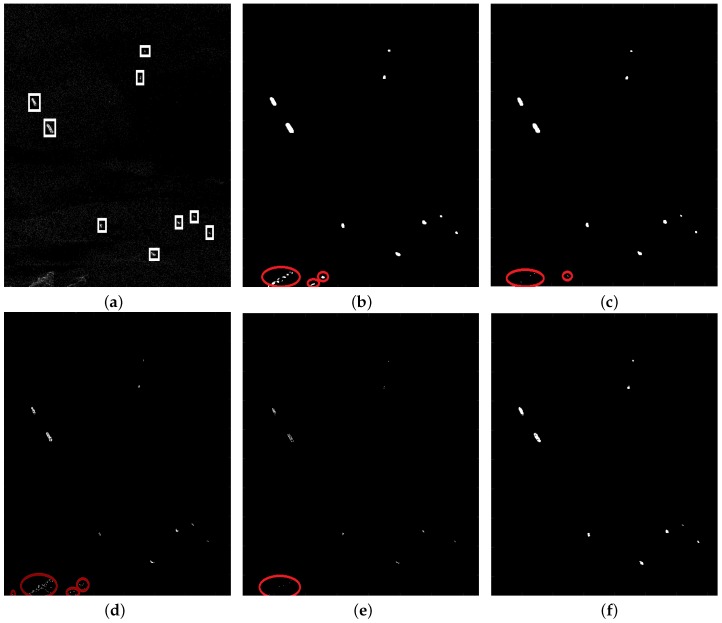
The scene with islands: (**a**) Original SAR image; (**b**) Detection result of VWIE; (**c**) Detection result of SVWIE; (**d**) Detection result of GΓD-CFAR; (**e**) Detection result of TS-CFAR; (**f**) Detection result of the proposed method.

Figure 8a is an image containing two small islands and nine ships (1932 × 1487 pixels). Figure 8b–f show detection results of VWIE, SVWIE, GΓD-CFAR, TS-CFAR and the proposed method, respectively. Because islands have strong backscattering as ships, VWIE, SVWIE, GΓD-CFAR and TS-CFAR mistakenly detect small islands as ships. However, the proposed method detects nine ships without false alarms. This is because the proposed SVWIE helps enhance ships while noncircularity helps to reduce or even eliminate the influence of strong backscattering of islands.

**Figure 9:**
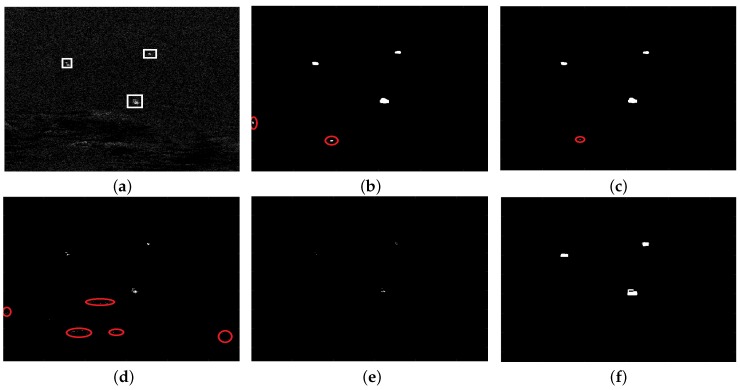
The heterogeneous scene: (**a**) Original SAR image; (**b**) Detection result of VWIE; (**c**) Detection result of SVWIE; (**d**) Detection result of GΓD-CFAR; (**e**) Detection result of TS-CFAR; (**f**) Detection result of the proposed method.

Figure 9a is an image containing three ships in the heterogeneous background (873 × 576 pixels). Figure 9b–f show detection results of VWIE, SVWIE, GΓD-CFAR, TS-CFAR and the proposed method, respectively. Because high speed wind causes fierce sea surface, backscattering of surrounding ocean is raised. Therefore, GΓD-CFAR has a bad performance in this case. VWIE could reduce most sea clutter, but have two false alarms. SVWIE further helps to suppress clutter, but there still remains one false alarm. TS-CFAR could detect three ships without false alarms but the detection results of ships are intermittent, because the ships in the original image Figure 9a are intermittent. However, the proposed method could enhance ships against the sea clutter and extract ships from complex background correctly.

**Figure 10:**
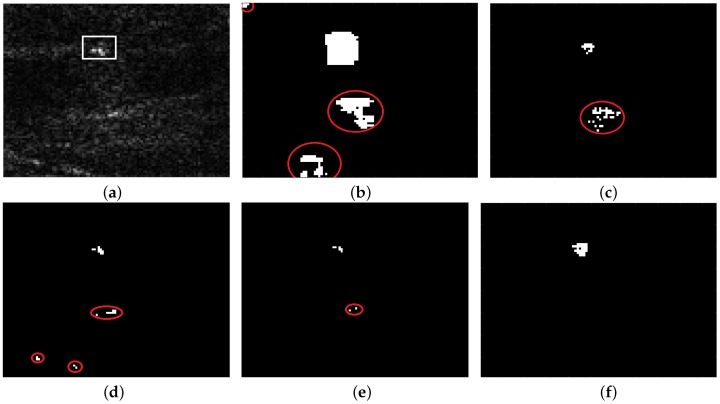
The scene with strong sea clutter: (**a**) Original SAR image; (**b**) Detection result of VWIE; (**c**) Detection result of SVWIE; (**d**) Detection result of GΓD-CFAR; (**e**) Detection result of TS-CFAR; (**f**) Detection result of the proposed method.

Figure 10a is an image containing one ships with strong noise (93 × 120 pixels). Figure 10b–f show detection results of VWIE, SVWIE, GΓD-CFAR, TS-CFAR and the proposed method, respectively. In the case, the clutter is quite interfering for detection. VWIE, SVWIE, GΓD-CFAR and TS-CFAR wrongly detect the strong clutter as ships. However, the clutter has low noncircularity, and therefore the proposed method could enhance the ship against the clutter and extract the ship from complex background correctly.

The figure-of-merit (FoM) [35] is used to evaluate the performance of different methods, which is defined as

(12) $$FoM=N_{tt}/\left(N_{fa}+N_{gt}\right),$$

where $N_{tt}$ is the number of correctly detected targets, $N_{fa}$ is the number of false alarms, and $N_{gt}$ is the number of true targets. Higher value of FoM means lower alarms and higher detection rate. The FoMs of aforementioned five methods are listed in Table 2. It is clear that the proposed method yields best performance compared to VWIE, SVWIE, GΓD-CFAR and TS-CFAR in the four different scenes. For Figure 8, Figure 9 and Figure 10, *h* detector can effectively reduce background obstruction and enhance ships, which is significant for detection.

## 4. Conclusions

A new adaptive ship detection method based on the SN decomposition is proposed for single-look complex SAR images. To take advantage of phase information, noncircularity level of SAR images is fully analyzed and used. Moreover, SVWIE is proposed by adding similarity measure into VWIE. According to SVWIE and noncircularity, we develop *h* detector based on SN decomposition, which can enhance ships and suppress background noise. The GΓD is suitable for the characterization of the *h* statistics of clutter and the CFAR technique based on *h* detector is proposed. Experimental results show that the proposed method achieves satisfying detection results in complex background compared to VWIE, SVWIE, GΓD-CFAR and TS-CFAR algorithms.

## Figures and Tables

**Table 1:** Quantitative evaluation of fitting results using the GΓD.

Patch	A	B	C	D
KL value	0.0007	0.0022	0.0003	0.0001

**Table 2:** FOMs of aforementioned five different methods.

	VWIE	SVWIE	GΓD-CFAR	TS-CFAR	the Proposed Method
Figure 7	1	1	1	1	1
Figure 8	0.75	0.82	0.69	0.90	1
Figure 9	0.60	0.75	0.38	1	1
Figure 10	0.25	0.50	0.25	0.50	1
